# Anaphylaxis triggered by rennet flower (*Withania coagulans*): A cautionary tale from traditional medicine

**DOI:** 10.1016/j.jacig.2026.100640

**Published:** 2026-01-12

**Authors:** Jefferson Daniel, Sekar Rajasekar, Devasahayam Jesudas Christopher

**Affiliations:** Department of Pulmonary Medicine, Christian Medical College, Vellore, India

**Keywords:** *Withania coagulans*, rennet flower, anaphylaxis, herbal medicine allergy, IgE-mediated anaphylaxis, traditional medicine allergy, Solanaceae allergy

## Abstract

This case report highlights the first documented instance of anaphylaxis caused by *Withania coagulans*, a natural remedy widely used in traditional medicine. Given the popularity of *W coagulans* among Asian communities worldwide, the report’s findings carry essential global clinical relevance.

The rennet flower, scientifically known as *Withania coagulans*, is a medicinal plant that has been utilized for centuries in the traditional Unani and Ayurveda medical systems. A member of the genus *Withania* and the family Solanaceae, it is predominantly found in India, Pakistan, and Afghanistan.^1^ Allergic reactions to members of the family Solanaceae are well documented. The plants contain alkaloids that can cause intolerance reactions. IgE-mediated hypersensitivity to tomato, potato, eggplant, and bell pepper has been reported, manifesting as oral allergy syndrome, urticarial rash, and (in rare cases) anaphylaxis.^2^ *Withania somnifera* (Ashwagandha), a close relative of *W coagulans*, is globally recognized for its adaptogenic and therapeutic properties, including anti-inflammatory, hypoglycemic, hepatoprotective, and antimicrobial effects.^3^^,^^4^ Both plants share a rich phytochemical profile, particularly with anolides, which contribute to their use in integrative medicine.^3^ Although *W* *coagulans* is less well known, it plays a prominent role within traditional medicine systems, especially for treatment of diabetes.^5^ Similar to quinine, which was discovered in cinchona bark, *W coagulans* may hold promise for the development of future allopathic treatments.^5^ This report presents what appears to be the first case of anaphylaxis in response to *W coagulans* to be documented in the medical literature. Informed consent for publication of these findings was obtained from the patient.

A 65-year-old man presented to the emergency department with generalized pruritus, facial and lip swelling, bilateral wheezing, and lightheadedness. On arrival, his blood pressure was 100/70 mm Hg, but it rapidly dropped to 90/60 mm Hg and became unrecordable. He was immediately triaged to the high-priority zone for emergency management. Inquiry with his family revealed that he had ingested a homemade decoction prepared from dried rennet flower (*W coagulans*) approximately 30 minutes before symptom onset. He had been diagnosed with diabetes mellitus during a recent routine evaluation. As a staunch proponent of natural remedies, he declined allopathic treatment. He consulted a local indigenous practitioner who recommended a rennet flower decoction for glycemic control. He had first consumed the decoction 2 weeks before the emergency department visit, approximately 2 hours after breakfast (rice pancakes with coconut chutney and tea with milk). Within 60 minutes of that first exposure, he had developed a generalized urticarial rash that resolved spontaneously. On the day of presentation, presuming the previous reaction to be due to overdose, he consumed half the previous dose approximately 30 minutes before symptom onset. This second exposure resulted in the severe anaphylactic symptoms described earlier in this report. The patient had no history of drug allergies or comorbidities (other than diabetes mellitus) and was not taking any medication. A working diagnosis of grade III anaphylaxis, per the World Allergy Organization classification, was made. The patient was promptly treated with an initial intramuscular epinephrine dose of 0.5 mL (in a concentration of 1:1000). Because of an inadequate response, 2 additional doses were given at 5-minute intervals, for a total of 3 doses. He also received intravenous pheniramine, hydrocortisone, fluids, and nebulized bronchodilators. Subsequently, a serum mast cell tryptase level obtained within 2 hours of the episode was elevated at 18.4 μg/L (vs a baseline level of 5.2 μg/L obtained 1 week later [reference range 2.00-14.00 μg/L]), supporting the diagnosis of anaphylaxis. The patient improved clinically and was admitted for observation to monitor for biphasic reactions. He was discharged after 24 hours of monitoring.

Four weeks later, the patient returned to the outpatient clinic for further evaluation. A skin prick test (SPT) was performed, yielding a positive result for rennet flower extract, with a 10 × 8-mm wheal and pseudopods (Fig 1). The negative control (normal saline) showed no response (0 mm), whereas the positive control (histamine) produced a 6-mm wheal. An SPT with the extract on one of the investigators (a nonallergic control) showed no reaction, excluding irritant effects and confirming allergen specificity.

**Fig 1:**
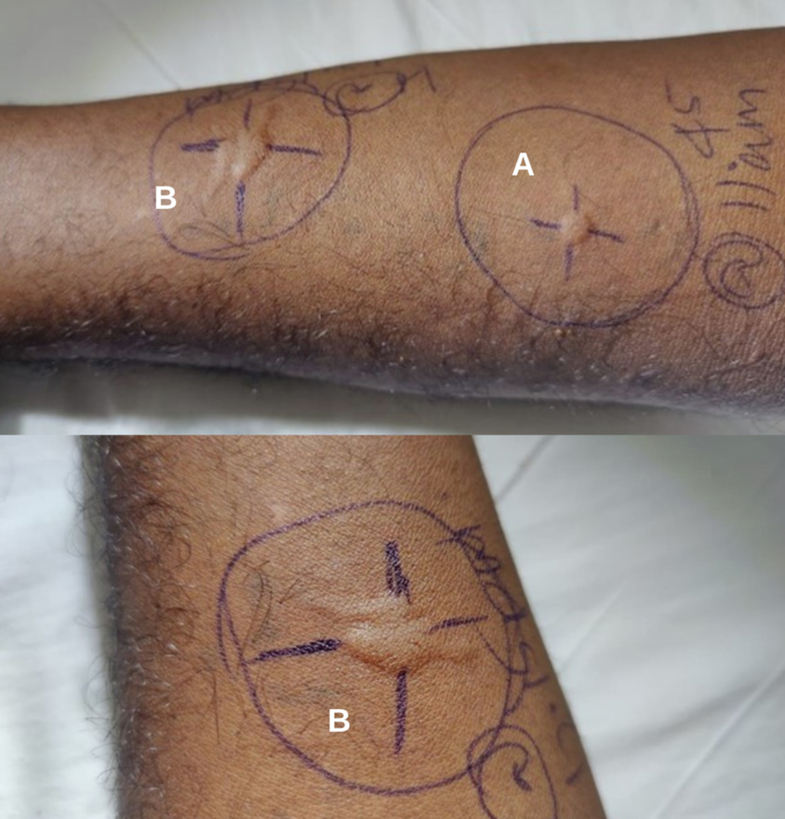
Photographs of the positive result of an SPT to *W coagulans* (rennet flower). Photograph showing a smaller (6 × 6-mm) circular wheal (the histamine control) (**A**) and a larger (10 × 8-mm) irregular wheal with pseudopod, indicating a positive reaction to *W coagulans* (**B**). The negative control (normal saline [not shown here]) showed no response [0 mm]).

On the basis of the patient’s clinical history, elevated acute-phase tryptase levels, and positive SPT findings, a diagnosis of IgE-mediated hypersensitivity to *W coagulans* was established. The patient was advised to avoid all herbal preparations containing *Withania* species. He was prescribed an emergency preloaded epinephrine injector and along with education on its use, and comprehensive anaphylaxis education was given. As of the time of preparation of this case report, a teleconsultation had confirmed that the patient had had no further allergic reactions or anaphylaxis for 3 years. Following this severe reaction, the patient completely discontinued all herbal and traditional medicine preparations.

Although rennet flower (*W coagulans*) has been used in traditional medicine, there is limited scientific literature documenting adverse reactions associated with it. To our knowledge, this is the first documented case of anaphylaxis triggered by this plant's flower. Despite the widespread use of rennet flower, particularly as an alternative treatment for diabetes, our patient appears to have experienced the first documented case of IgE-mediated anaphylaxis in response to *W coagulans*, highlighting a previously unrecognized allergenic potential.^5^ In India, there is increasing evidence of allergic reactions linked to Ayurvedic and herbal medicines, underscoring the need for vigilance.^6^^,^^7^ We aimed to identify the specific allergenic protein using immunoblotting and mass spectrometry; however, the patient declined repeat sample submission. This approach would have identified the molecular culprits, which are likely to be withanolides, lipid transfer proteins, profilins, or pathogenesis-related proteins commonly implicated in plant allergies. Molecular characterization would have enabled prediction of cross-reactivity patterns within the Solanaceae family and with the more widely used W somnifera.

Identifying the cause of anaphylaxis is often challenging, and the rarity of IgE-mediated reactions to members of the Solanaceae family adds complexity. Our findings are especially relevant for allergy practitioners in India and other countries in which patients of South Asian origin often receive traditional remedies with standard allopathic care.^8^^,^^9^ This case documents a novel trigger for anaphylaxis in a plant that is widely used in traditional medicine. Identifying specific allergens, even rare ones, has scientific value: it adds another differential diagnosis to consider in idiopathic anaphylaxis cases, particularly among patients using herbal remedies. As use of traditional medicine continues to increase globally and is often undisclosed to physicians, systematic documentation of such reactions contributes to reducing unexplained anaphylaxis and improving patient safety. *W coagulans* is not approved as a pharmaceutical drug; however, it is widely available as dried plant material in traditional medicine markets and may be sold as a dietary supplement or food additive with minimal regulatory oversight. This lack of standardization complicates allergen avoidance and risk assessment.

## Disclosure statement

Disclosure of potential conflict of interest: The authors declare that they have no relevant conflicts of interest.
